# Negative Interplay between Biofilm Formation and Competence in the Environmental Strains of *Bacillus subtilis*

**DOI:** 10.1128/mSystems.00539-20

**Published:** 2020-09-01

**Authors:** Qianxuan She, Evan Hunter, Yuxuan Qin, Samantha Nicolau, Eliza A. Zalis, Hongkai Wang, Yun Chen, Yunrong Chai

**Affiliations:** a Department of Biology, Northeastern University, Boston, Massachusetts, USA; b Institute of Biotechnology, Zhejiang University, Hangzhou, China; University of Pittsburgh Medical Center

**Keywords:** biofilm, competence, *Bacillus subtilis*, cell differentiation, environmental strains

## Abstract

Environmental strains of the soil bacterium Bacillus subtilis have valuable applications in agriculture, industry, and biotechnology; however, environmental strains are genetically less accessible. This reduced accessibility is in sharp contrast to laboratory strains, which are well known for their natural competence, and a limitation in their applications. In this study, we observed that robust biofilm formation by environmental strains of B. subtilis greatly reduced the frequency of competent cells in the biofilm. By using model strain 3610, we revealed a cross-pathway regulation that allows biofilm matrix producers and competence-developing cells to undergo mutually exclusive cell differentiation. We further demonstrated that the competence activator ComK represses the key biofilm regulatory gene *sinI* by directly binding to the *sinI* promoter, thus blocking competent cells from simultaneously becoming matrix producers. In parallel, the biofilm activator SlrR represses competence through three distinct mechanisms involving both genetic regulation and cell morphological changes. Finally, we discuss the potential implications of limiting competence in a bacterial biofilm.

**IMPORTANCE** The soil bacterium Bacillus subtilis can form robust biofilms, which are important for its survival in the environment. B. subtilis also exhibits natural competence. By investigating competence development in B. subtilis *in situ* during biofilm formation, we reveal that robust biofilm formation often greatly reduces the frequency of competent cells within the biofilm. We then characterize a cross-pathway regulation that allows cells in these two developmental events to undergo mutually exclusive cell differentiation during biofilm formation. Finally, we discuss potential biological implications of limiting competence in a bacterial biofilm.

## Abstract (precis)

The soil bacterium Bacillus subtilis can form robust biofilms, which are important for its survival in the environment. B. subtilis also exhibits natural competence. By investigating competence development in B. subtilis *in situ* during biofilm formation, we reveal that robust biofilm formation often greatly reduces the frequency of competent cells within the biofilm. We then characterize a cross-pathway regulation that allows cells in these two developmental events to undergo mutually exclusive cell differentiation during biofilm formation. Finally, we discuss potential biological implications of limiting competence in a bacterial biofilm.

## INTRODUCTION

**FIG 1:**
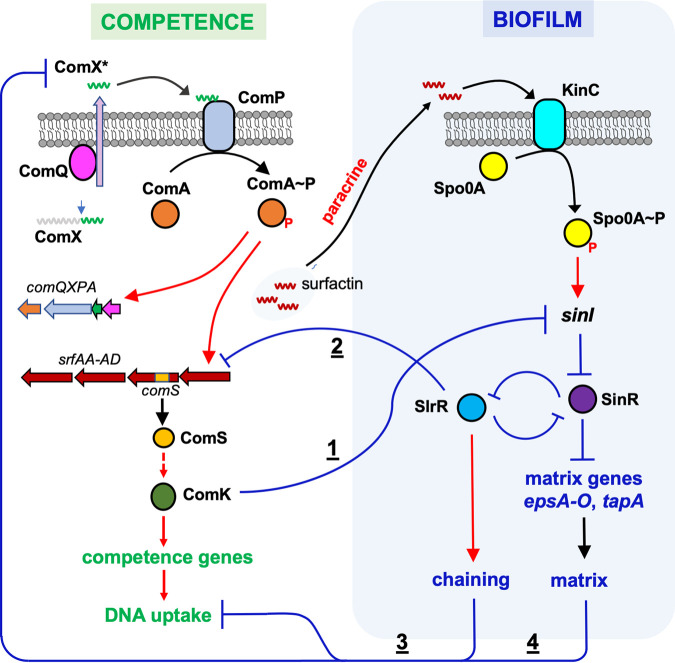
Working model for cross-pathway regulation between competence and biofilm in B. subtilis. Competence development is initiated when the quorum-sensing (QS) peptide derived from ComX (ComX*) is sensed by a membrane histidine kinase ComP (36). The response regulator ComA then activates an *srfAA-AD* operon and an embedded small gene, *comS*. The latter encodes a positive regulator, ComS, for the competence activator, ComK (41). ComK^ON^ cells express late competence genes, which ultimately differentiate into competent cells ready for DNA uptake. Here, we propose that ComK simultaneously and negatively regulates the biofilm pathway by repressing the key biofilm regulatory gene *sinI* (shown as 1). SinI antagonizes the biofilm master repressor SinR to derepress genes for the biofilm matrix production (*epsA-O*, *tapA*, etc.). Negative regulation of *sinI* by ComK is expected to inhibit biofilm formation. SlrR is another antagonist of SinR that forms a double-negative loop with SinR. Under biofilm-inducing conditions, *sinI* is activated by the developmental master regulator Spo0A in response to sensory kinases (e.g., KinC) sensing various environmental signals. Here, we also propose that the biofilm regulator SlrR negatively regulates competence development through several distinct mechanisms. First, SlrR activates matrix production, which physically blocks sensing of the quorum-sensing peptide signal ComX* (shown as 4) (49); second, SlrR-induced cell chaining may block DNA uptake since DNA uptake machinery was shown to be pole-localized (57, 58) (shown as 3); and third, SlrR negatively regulates the *srfAA-AD* operon and *comS* (shown as 2). Red arrows and blue lines represent positive and negative regulation, respectively. ComX*, a secreted QS peptide derived from ComX. Surfactin induces matrix production by a paracrine signaling mechanism (49).

Bacillus subtilis is a soil-dwelling, spore-forming bacterium that is widespread in nature. This microorganism is also a plant growth-promoting rhizobacterium (PGPR) and an excellent biological control agent with various beneficial activities in plant protection (1). Biological control by B. subtilis is attributed to a number of important abilities of the bacterium, including antibiotic production, inhibition of pathogenic fungi and parasites, induction of plant systemic resistance, and formation of plant root-associated biofilms (2–4). Biofilms are communities of microorganisms that are encased in a self-produced extracellular matrix, which provides protection to cells in the biofilm against various biotic and abiotic stresses (5–8). Undomesticated strains of B. subtilis form robust biofilms in response to a variety of environmental cues and cellular signals (9–16). Some of those strains, such as NCIB3610 (here referred to as 3610), are used as models to study bacterial biofilm formation (2, 14, 17–20). Biofilm formation by B. subtilis is initiated by one or several histidine kinases (KinA-KinD) that sense environmental signals and activate a phosphorelay (Spo0F-Spo0B-Spo0A), which then activates Spo0A, a master regulator of sporulation and biofilm formation, via protein phosphorylation (Fig. 1) (21, 22). Phosphorylated Spo0A (Spo0A∼P) activates *sinI*, and the protein SinI is an antagonist that counteracts the biofilm repressor SinR (23, 24). Another regulatory protein, SlrR, is also involved in antagonizing SinR (25). SlrR and SinR form a double-negative regulatory switch (26). Under nonbiofilm conditions, the matrix operons are tightly repressed. Interestingly, domesticated strains of B. subtilis that have been utilized in laboratory researches for many decades because of their easy genetic manipulation have lost their ability to form robust biofilms due to genetic mutations that have occurred during domestication (19, 20, 27).

Cell differentiation is a hallmark feature in bacterial biofilm development. In B. subtilis, it is known that cells in the biofilm differentiate into phenotypically distinct cell types (2, 14, 28). Some of these cell types may overlap or become mutually exclusive. For example, previous studies showed that a subpopulation of cells became sessile matrix producers, while cells in another subpopulation remained motile (26, 29). These two types of cells are mutually exclusive because of control by an epigenetic switch involving several regulatory genes, including *sinR*, *slrR*, *sinI*, and *ymdB* (26, 30–32). The switch allows bifurcation of the population into SlrR^ON^ and SlrR^OFF^ cells, which correspond to sessile and motile cells, respectively, because of SlrR-mediated gene regulation (26, 32). Some cells in the B. subtilis biofilm undergo sporulation (19). Spores often constitute 15 to 20% of the total cell population in a mature B. subtilis biofilm. It has been proposed that Spo0A∼P initially accumulates to intermediate levels in matrix producers (33). When the levels of Spo0A∼P keep rising in those matrix producers, they eventually activate the expression of hundreds of genes involved in sporulation (34, 35). This may explain how matrix producers transition to become sporulating cells and why sporulating cells, as a cell type, are inclusive to matrix producers.

Natural competence is an ability of certain bacterial species to acquire environmental DNA for genetic exchange (36, 37). Natural competence is evolutionarily important to the development of increased genetic diversity and adaptability of bacteria. In B. subtilis, the complex regulatory network controlling competence has been elucidated (36–39). Competence is initiated when cells produce a peptide pheromone derived from ComX (Fig. 1), which is also a quorum-sensing molecule (40). This quorum-sensing peptide is sensed on the membrane by a sensory kinase ComP of the ComA-ComP two-component system (36). The response regulator ComA then activates a *srfAA-AD* operon, which not only encodes enzymes involved in the biosynthesis of surfactin but also transcribes a small gene, *comS* (41). The protein ComS is critical to the activation of the competence activator ComK by releasing ComK from MecA, an adaptor protein that normally routes ComK to proteolysis (42, 43). Hence, the presence of ComS allows ComK to accumulate. In addition, the *comK* gene is subject to a bistable control mechanism that ensures only a subset of cells accumulate high levels of this competence activator and enter the so-called K-state (38). Unlike well-studied laboratory strains, many environmental strains of B. subtilis are much less competent for reasons that are still unclear. In the model strain 3610, a *comI* gene located in a cryptic plasmid was shown to inhibit the competence, while the deletion of this gene or curing of the cryptic plasmid boosted transformation efficiency by >100-fold (44). However, it is not known whether such plasmid-born competence inhibitory genes are broadly present in environmental strains. Another regulatory gene, *degQ*, was also shown to reduce competence in strain 3610, and the deletion of *degQ* similarly boosted transformation efficiency of 3610 (27). There is also evidence that a point mutation identified in the promoter of *degQ* in laboratory strain 168, which likely lowers the expression of *degQ*, contributes to the much higher transformation efficiency of strain 168 (27). Although competence-developing cells are believed to be present in B. subtilis biofilms (28), competence development has not been thoroughly investigated in the context of the biofilm (45).

In this study, we investigated factors that impacted competence in environmental strains of B. subtilis. We found an interplay between biofilm formation and competence development in which robust biofilm formation in the environmental strains greatly reduces the frequency of competent cells within the biofilm. Furthermore, we show that a very low number of cells express a late competence gene reporter and that those cells become mutually exclusive from matrix producers in the biofilm. Finally, we characterize a cross-pathway regulation that contributes to the above mutual exclusivity and limits competence in individual cells in the biofilm.

## RESULTS

### *B. subtilis* environmental strains are robust biofilm formers but poor in competence.

**FIG 2:**
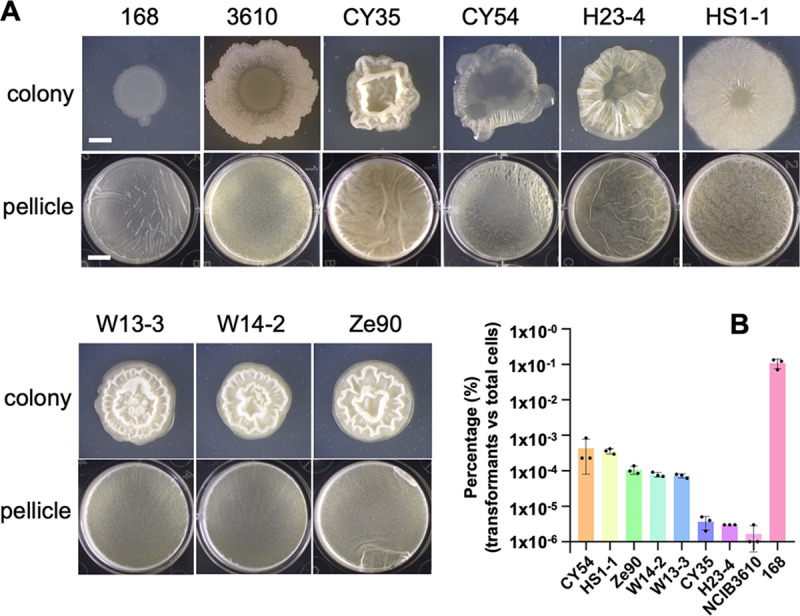
B. subtilis environmental strains are strong biofilm producers but have poor competence. (A) Colony and pellicle biofilm phenotypes of seven environmental strains of B. subtilis plus strains 168 and 3610. Scale bar in the picture of the colony, 2 mm; scale bar in the picture of the pellicle, 5 mm. The scale bar in the picture of colony applies to all pictures of colonies, as does the scale bar in the picture of the pellicle. (B) Transformation efficiency of the seven environment isolates of B. subtilis plus strains 168 and 3610. The results are shown as percentages of the number of transformants relative to the total numbers of cells. Assays were performed in triplicates. Each dot represents one technical replicate. Error bars represent the standard deviations.

**FIG 3:**
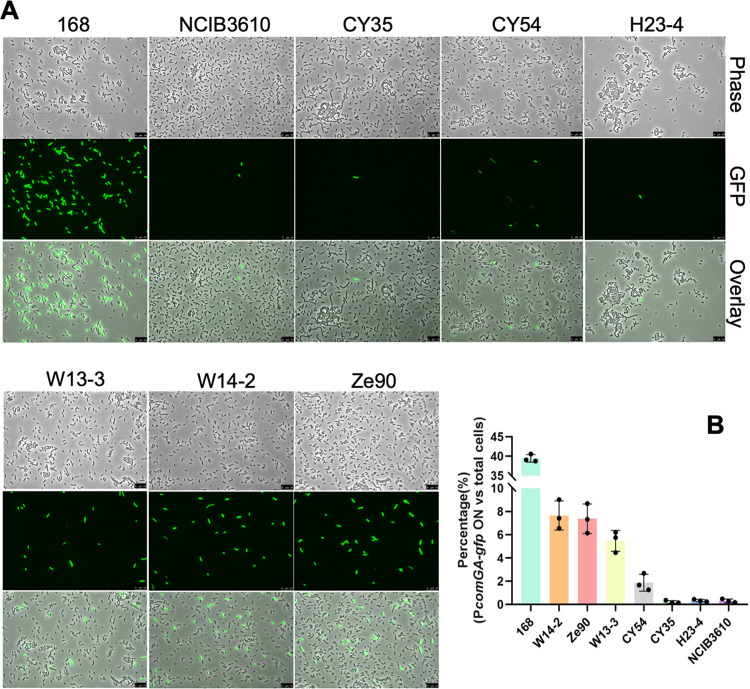
A small proportion of cells from the environmental strains express the late competence gene *comGA*. (A) Environmental strains harboring the late competence gene reporter P*_comGA_*-*gfp* were grown in the competence medium (MC) to the early stationary phase. Cells were harvested and observed under fluorescence microscopy. The 168 and 3610 strains are included for comparison. Representative images are shown here. Scale bars, 10 μm. (B) Percentages of P*_comGA_*-*gfp*-expressing cells relative to the total number of cells in seven different environmental strains plus strains 168 and 3610. In each bar, the three dots represent three individual data points calculated from three different images consisting of about 600 to 800 cells in total per sample. Error bars represent the standard deviations.

Previously, we investigated a number of environmental isolates of B. subtilis for their biological control activities in plant protection (4). Many of these strains form robust biofilms in biofilm-inducing media (Fig. 2A) and on plant roots (4, 10). However, those environmental strains are much more difficult to manipulate genetically than laboratory strains. Specifically, they show a much lower transformation efficiency that is hundreds to tens of thousands of times lower than that of the laboratory strain 168 (Fig. 2B). In these tested strains, large variations in transformation efficiency were also seen. For example, there was a several 100-fold difference in transformation efficiency between 3610 and CY54 based on the percentage of transformants relative to the total number of cells (Fig. 2B). To test whether variations in transformation efficiency were due to altered competence gene regulation, we constructed a fluorescent reporter for a late-stage competence gene, *comGA* (P*_comGA_*-*gfp*), and introduced the reporter into the environmental strains, as well as strain 168. The engineered reporter strains were grown in competence medium (MC) to the early stationary phase, at which time cells were examined under fluorescence microscopy. P*_comGA_*-*gfp*-expressing cells were observed, but only in a small subset of cells (with the exception of strain 168, Fig. 3A). We also noticed a clear bimodal pattern in P*_comGA_*-*gfp* expression, indicating that bistability in competence development previously reported in laboratory strains is also reinforced in environmental strains (38). The ratio of P*_comGA_*-*gfp*-expressing cells in different environmental strains varied significantly and ranged from about 0.25 to 7.7%, which was much lower than that in strain 168 (∼40%, Fig. 3B). In general, the results of assays of the fluorescent reporter correlated well with those from genetic transformation, with the exception of CY54 (Fig. 2B and 3B), suggesting that the reduced transformation efficiency in environmental strains is likely due to altered genetic regulation. In summary, the environmental strains are robust biofilm formers but have poor competence.

### DegQ negatively impacts competence in some, but not all, tested environmental strains.

Previous studies suggested that the *degQ* gene negatively regulates genetic competence in B. subtilis (27). In the present study, a deletion mutation in *degQ* increased the transformation efficiency of 3610 by a few folds, while *degQ* overexpression led to a reduction in transformation efficiency (see Fig. S1A in the supplemental material). DegQ is believed to impact competence through DegU, a response regulator and a transcription factor on the *comK* gene. A *degU* deletion mutation almost completely eliminated competence in 3610, while the deletion mutation of *degS*, which encodes the histidine kinase of the DegS-DegU two-component system (46), modestly impaired the transformation efficiency (see Fig. S1A). To test whether DegQ plays a similar role in competence in the environmental strains, we introduced Δ*degQ* into these strains and examined the transformation efficiency of the resulting mutants. Δ*degQ* increased transformation efficiency in five of the seven tested strains (all except for Ze90 and W13-3) by ∼2- to ∼88-fold compared to the corresponding wild-type strains (see Fig. S1B). In Ze90 and W13-3, Δ*degQ* decreased the transformation efficiency by ∼4- and ∼7-fold, respectively (see Fig. S1B). These results indicate that the impact of DegQ on competence varies in different environmental strains.

10.1128/mSystems.00539-20.1 FIG S1 DegQ impacts competence negatively in some, but not all, environmental strains. (A) Transformation efficiency of wild-type, Δ*degQ*, *degQ* overexpression, Δ*degS*, and Δ*degU* strains of B. subtilis 3610. The results are shown as the percentage of the number of transformants versus the number of total cells. Assays were done in triplicates. Error bars represent the standard deviations. *, *P* < 0.05. A *t* test was used to perform statistical analysis. (B) Comparison of transformation efficiency between the Δ*degQ* deletion mutants and the respective wild-type environmental strains. Numbers in the *y* axis represent fold changes comparing CFU counts of the Δ*degQ* deletion mutants and those of the respective wild-type strains. Assays were done in triplicates. Error bars represent the standard deviations. (C) DNA sequence alignments of the *degQ* promoter region from seven environmental strains plus strains 168 and 3610. Newly identified nucleotide changes in environmental strains, which correspond to the −28, −48, and −77 positions relative to the *degQ* transcription start, are highlighted in red boxes. The previously identified single nucleotide change in strain 168 is highlighted in the blue box. Download FIG S1, TIF file, 2.4 MB. Copyright © 2020 She et al. 2020 She et al. This content is distributed under the terms of the Creative Commons Attribution 4.0 International license.

Finally, we suspected that the same or similar point mutation identified in the *degQ* promoter in laboratory strain 168 is present in any of those environmental strains. We amplified the promoter region of the *degQ* gene by PCR and conducted DNA sequencing, which revealed that the point mutation in *degQ* in strain 168 was not present in any of the environmental strains (highlighted in the blue square; see Fig. S1C). However, additional mutations were identified in the promoter of *degQ* in CY35 and CY54 (single-nucleotide changes at positions −28, −48, and −77 from the *degQ* transcription start, highlighted in red squares; see Fig. S1C). These newly identified mutations have not yet been tested to determine whether they impact *degQ* expression and competence in CY54 and CY35.

### Matrix producers and competent cells are mutually exclusive in the *B. subtilis* biofilm.

**FIG 4:**
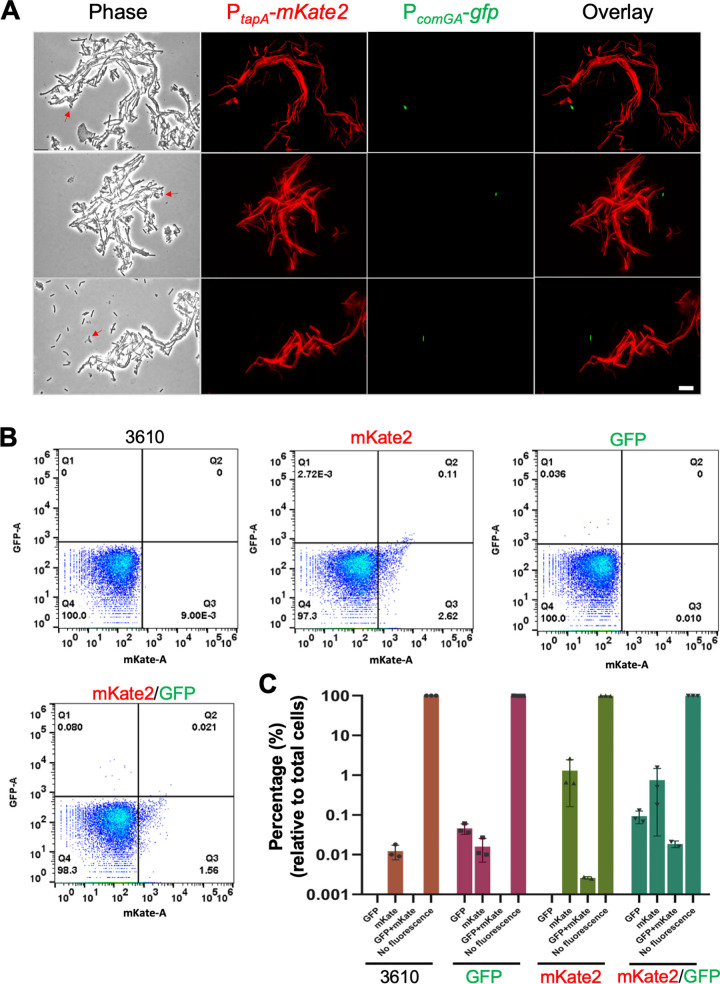
Matrix producers and competent cells are mutually exclusive in the strain 3610 biofilm. (A) Fluorescent microscopic analyses of cells collected from a B. subtilis 3610 pellicle biofilm bearing dual fluorescent reporters of P*_comGA_*-*gfp* and P*_tapA_*-*mKate2* (EH43). The activity of P*_tapA_*-*mKate2* (cells in red) indicates expression of the key biofilm matrix operon *tapA-sipW-tasA*, while P*_comGA_*-*gfp* reports a late competence gene *comGA* (cells in green). More images are available in Fig. S2 in the supplemental material. Scale bar, 10 μm. The scale bar is representative of all images in the figure. (B) Flow cytometry analyses of the above dual fluorescent reporter strain (EH43, indicated as mKate2/GFP), two single reporter strains (QS34 for P*_comGA_*-*gfp* and EH41 for P*_tapA_*-*mKate2*, indicated as GFP and mKate2, respectively), and strain 3610 (as a gating control). The activities of P*_comGA_*-*gfp* and P*_tapA_*-*mKate2* were measured in GFP (*y* axis) and RFP (for mKate2, *x* axis) filters, respectively. Numbers represent the percentages of gated cells versus the total cells in the corresponding quadrant. (C) Quadrant analyses of the flow cytometry results. The percentage indicates gated cells/total cells in the corresponding quadrant. Each dot represents one biological replicate. Experiments were repeated three times. Error bars indicate the standard deviations (one dot representing the mKate2/GFP quadrant from the single reporter [EH41, shown as GFP] and one dot representing the mKate2/GFP quadrant from the double reporter [EH43, shown as mKate2/GFP] were omitted due to errors).

To understand why environmental strains have poor competence, we examined competence development *in situ* during biofilm formation in strain 3610. A dually labeled fluorescent reporter strain of 3610 (P*_tapA_*-*mkate2* and P*_comGA_*-*gfp*, EH43) was constructed, which allowed us to measure the expression of the matrix operon *tasA-sipW-tapA* (P*_tapA_*-*mKate2*) and the activity of the late-stage competence gene *comGA* (P*_comGA_*-*gfp*) simultaneously in the same cells (47, 48). Cells from a 3-day pellicle biofilm by the dual-reporter strain were collected and examined under fluorescence microscopy. Cells were seen in bundled chains and showed strong expression of the matrix reporter P*_tapA_*-*mKate2* (chaining and matrix production are known to be coregulated during biofilm development [32]) (Fig. 4A; see also Fig. S2). In contrast, cells expressing P*_comGA_*-*gfp* were very rare, always in singlets, and almost never overlapped with cells expressing P*_tapA_*-*mKate2*. Because the number of cells expressing P*_comGA_*-*gfp* was very low, flow cytometry was applied to quantitatively determine the ratio of cells expressing the two reporters in the pellicle biofilm. Cells were similarly collected, treated with mild sonication to disrupt the bundled chains, and subjected to flow cytometry. As shown in Fig. 4B and C, only 0.08% of the total cells expressed P*_comGA_*-*gfp* (the average results from three replicates are shown in Fig. 4C). The finding that the ratio of P*_comGA_*-*gfp*-expressing cells was even lower in this assay than in the assay depicted in Fig. 3B (∼0.25% for strain 3610) was likely because the assay depicted in Fig. 3 was conducted under conditions that favored competence (use of competence medium, cells collected at the early stationary phase, etc.). Moreover, the results shown here strongly suggest that in the B. subtilis biofilm, matrix producers and competent cells rarely overlap, indicating that they are mutually exclusive cell types (Fig. 4A and B; see also Fig. S2).

10.1128/mSystems.00539-20.2 FIG S2 Matrix producers and competent cells are mutually exclusive in the strain 3610 biofilm. Fluorescent microscopic analyses of cells collected from a B. subtilis 3610 pellicle biofilm bearing dual fluorescent reporters of P*_comGA_*-*gfp* and P*_tapA_*-*mKate2* (EH43). In the overlay images above, the activity of P*_tapA_*-*mKate2* (cells in red) indicates expression of the key biofilm matrix operon *tapA-sipW-tasA*, while P*_comGA_*-*gfp* reports a late competence gene *comGA* (cells in green). Scale bar, 10 μm. The scale bar is representative for all images in the figure. Download FIG S2, TIF file, 2.4 MB. Copyright © 2020 She et al. 2020 She et al. This content is distributed under the terms of the Creative Commons Attribution 4.0 International license.

### Overexpression of the competence activator gene *comK* blocks biofilm formation in *B. subtilis*.

**FIG 5:**
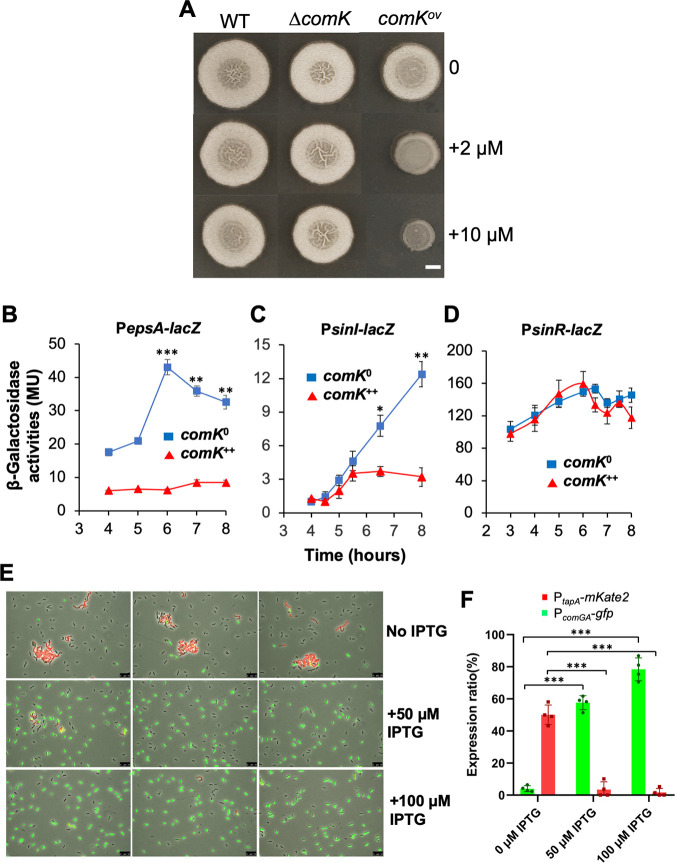
*comK* negatively regulates key biofilm genes. (A) Overexpression of *comK* impairs biofilm formation in B. subtilis. Phenotypes of the colony biofilms formed by the wild type (strain 3610), the Δ*comK* mutant (YC100), and the wild-type strain harboring an IPTG-inducible copy of *comK* (YC142) on MSgg plates supplemented with 0, 2, or 10 μM IPTG. Scale bar, 2 mm. The scale bar is representative for all pictures in this panel. (B to D) Wild-type strains bearing both an IPTG-inducible copy of *comK* and one of the three biofilm gene reporters—P*_epsA_-lacZ* (B, YC160), P*_sinI_-lacZ* (C, YC159), and P*_sinR_-lacZ* (D, YC177)—were assayed for β-galactosidase activities. Cells were cultured in MSgg with shaking in the absence (*comK*^0^) or presence (*comK*^++^) of 10 μM IPTG to induce *comK*. Assays were done in triplicate. Error bars represent the standard deviations. The *t* test was applied for statistical analysis. *, *P* < 0.05; **, *P* < 0.005; ***, *P* < 0.0005. (E) Fluorescent microscopic analyses of the dual reporter strain (P*_comGA_*-*gfp* and P*_tapA_*-*mKate2*) that also contains an IPTG-inducible copy of *comK* (EH44). Cells were grown with shaking MSgg to log phase (OD_600_ = 0.5), split into three fractions (one without IPTG and the other two with either 50 or 100 μM IPTG), and cultured for an additional hour before being harvested and analyzed by fluorescence microscopy. Scale bars, 10 μm. (F) Quantitative analyses of the dual reporter activities upon *comK* overexpression. For each IPTG concentration (0, 50, or 100 μM), the individual dots represent results from four separate images (in one biological replicate) consisting of about 600 to 800 cells in total. Error bars represent the standard deviations. A *t* test was used to perform statistical analysis. ***, *P* < 0.0005.

We hypothesized that the biofilm and the competence pathways negatively cross-regulate each other, resulting in these two cell types becoming mutually exclusive. To test this hypothesis, we first looked at the competence pathway. Since ComA-ComP is also important for biofilm formation (49), we focused on the competence activator, ComK, which acts downstream of ComA-ComP in competence development (Fig. 1) (43). A *comK* deletion mutation was constructed, and the biofilm phenotype of the mutant examined. Surprisingly, the mutant did not show any noticeable biofilm phenotype compared to the wild type (Fig. 5A). Given the low ratio of cells expressing P*_comGA_*-*gfp*, we reasoned that ComK was not active in the majority of cells, which would explain why the *ΔcomK* mutant did not differ from the wild-type strain in the biofilm phenotype. We then tested *comK* overexpression using an IPTG (isopropyl-β-d-thiogalactopyranoside)-inducible copy of *comK* that was constructed and introduced into strain 3610. The engineered strain displayed a strong biofilm defect upon the addition of IPTG (Fig. 5A), indicating that ComK strongly impacts biofilm development in B. subtilis. ComK activation is known to eventually cause growth arrest in B. subtilis (50). As an important control, the growth of the *comK* overexpression strain was examined. Upon induction of *comK* in the presence 10 μM IPTG, no difference in the growth rates of the cells was found compared to without *comK* induction (see Fig. S3A in the supplemental material).

10.1128/mSystems.00539-20.3 FIG S3 (A) Mild overexpression of *comK* does not cause growth inhibition. The wild-type strain harboring an IPTG-inducible copy of *comK* (YC142) was inoculated in MSgg broth and grown at 37°C over a period of 10 h. IPTG was either not added (*comK*^0^) or added to the medium at the final concentration of 10 μM to induce mild *comK* overexpression (*comK*^++^). Cell were periodically collected, and the cell optical density (OD_600_) was measured. (B) Comparison of transformation efficiency between the *epsA-O* mutants of the environmental strains and their respective wild-type strains. The results are presented in log scale as the percentage of the number of transformants versus the number of total cells. Experiment was done in triplicates. Error bars represent the standard deviations. *, *P* < 0.05; ***, *P* < 0.0005. A *t* test was used to perform statistical analysis. Download FIG S3, TIF file, 2.4 MB. Copyright © 2020 She et al. 2020 She et al. This content is distributed under the terms of the Creative Commons Attribution 4.0 International license.

### ComK negatively regulates biofilm matrix genes.

To further characterize the impact of ComK on biofilm formation, we tested whether ComK regulates any matrix genes, such as the *epsA-O* or *tapA* operons. A previously constructed transcription reporter P*_epsA_*-*lacZ* was introduced into the *comK* overexpression strain (51), and the resulting strain (YC160) was used to test the impact of *comK* overexpression on the activity of P*_epsA_*-*lacZ.* As shown in Fig. 5B, the activity of P*_epsA_*-*lacZ* decreased dramatically upon the addition of 10 μM IPTG to induce *comK*. In another test, we introduced a similar *comK* overexpression construct (*thrC*::P*_spank_*-*comK*) into the above-described dual fluorescent reporter strain (P*_tapA_*-*mkate2* and P*_comGA_*-*gfp*). When the resulting strain (EH44) was grown in MSgg (monosodium glutamate-glycerol) without the addition of IPTG, about 50% of the cells expressed P*_tapA_*-*mkate2* (cells in red, Fig. 5E and F). Again, a very low number of cells expressed P*_comGA_*-*gfp* (cells in green, Fig. 5E and F). Upon addition of 50 or 100 μM IPTG to induce *comK* expression for about an hour, the competence reporter P*_comGA_*-*gfp* was found activated in a significantly increased subpopulation of cells (about 58 or 78% of the total cells, middle and lower panels, respectively, Fig. 5E and F). In addition, the expression of P*_tapA_*-*mKate2* was turned off almost completely (cells in red, middle and lower panels, Fig. 5E and F). Moreover, matrix producers and competent cells rarely overlapped in this assay. Overall, our results suggest that ComK negatively regulates biofilm matrix genes.

### ComK negatively regulates *sinI*.

The *epsA-O* and the *tapA* operons are directly repressed by SinR, while derepression occurs when SinI counteracts SinR through protein-protein interactions (23, 24, 52, 53). To determine how ComK negatively regulates the matrix operons, we tested whether ComK regulates either *sinI* or *sinR*. We took a similar approach by applying previously constructed transcription reporters of P*_sinI_*-*lacZ* and P*_sinR_*-*lacZ* (51). Each reporter was introduced into the *comK* overexpression strain, and the impact of *comK* overexpression on the activity of the reporters was similarly tested. Indeed, *comK* overexpression had a strong negative impact on the activity of P*_sinI_*-*lacZ*, but not on P*_sinR_*-*lacZ* (Fig. 5C and D). These results indicate that ComK negatively regulates the matrix operons, likely through its regulation of *sinI*.

### ComK directly binds to the regulatory region of *sinI*.

**FIG 6:**
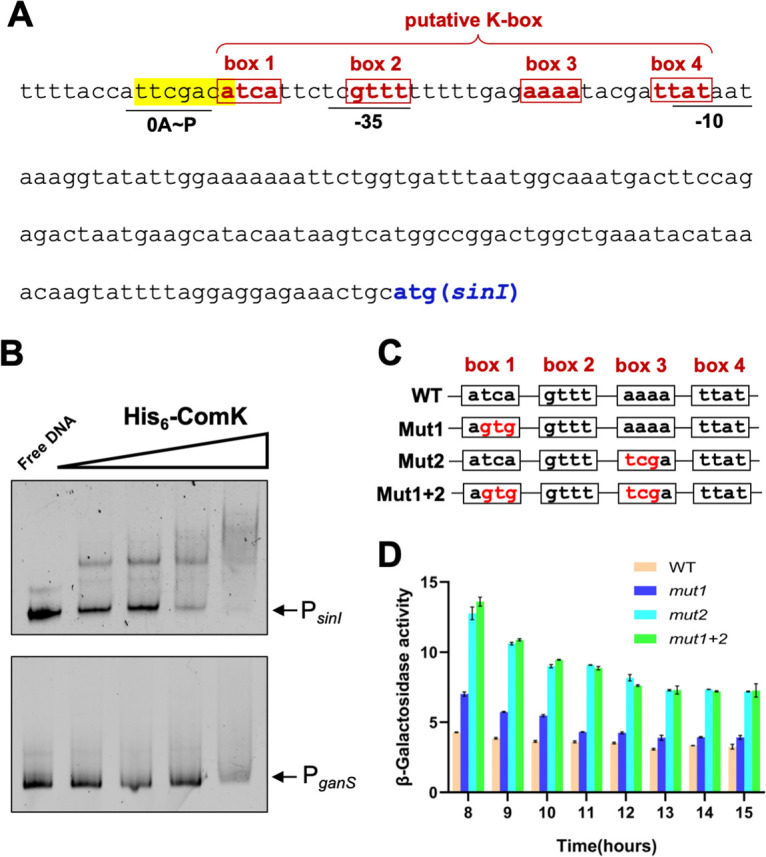
ComK directly binds to the promoter of *sinI.* (A) DNA sequence of the promoter region of *sinI*. The Spo0A∼P activation site (in yellow and underlined), the −10 and −35 motifs (underlined), and putative ComK boxes (from box 1 to 4) are highlighted (31). (B) EMSA of His_6_-ComK binding to the promoter of *sinI.* P*_sinI_* was end labeled with Cy3 dye and used as the DNA probe. Cy3-labeled P*_ganS_* was used as a negative control. The far-left lanes are the control of free DNA without proteins. A decreasing gradient of 150, 60, 15, and 7.5 nM recombinant His_6_-ComK was applied in the lanes in the EMSA as indicated. A total of 200 pmol of fluorescent-labeled DNA probe was applied in each lane. (C) Site-directed mutagenesis of the ComK boxes in the *sinI* promoter is indicated. Nucleotide changes in box 1 (mut1) and box 3 (mut2) are highlighted in red. Changes in box 2 and box 4 are avoided due to their overlap with −10 and −35 promoter motifs. (D) β-Galactosidase activities of the cells with an inducible *comK* construct and bearing either wild-type P*_sinI_-lacZ* or the reporter fusions with indicated point mutations in the K-box (mut1, mut2, and mut1 + 2, as shown in panel C) were performed. IPTG was added at a 10 μM concentration in the media. Cells were grown with shaking in MSgg. Samples were periodically collected and assayed for β-galactosidase activities. Assays were performed at least in triplicate. Error bars represent the standard deviations.

ComK regulates genes through binding to the so-called K-box, which is often found in the regulatory region of the genes (54). When the promoter sequence of *sinI* was analyzed, a region of DNA sequence that resembles the consensus K-box (“AAAA-N_5_-TTTT-N_8_-AAAA-N_5_-TTTT”) was recognized (Fig. 6A). This DNA sequence overlaps both the −35 and the −10 motifs of the Sigma A-dependent promoter and a Spo0A∼P activation site (OA∼P) in the *sinI* promoter (33). ComK binding to this putative K-box could prevent *sinI* transcription. An electronic mobility shift assay (EMSA) was performed to test whether the ComK protein binds to the promoter of *sinI*. Recombinant His_6_-ComK proteins were expressed in Escherichia coli and purified. Fluorescently end-labeled DNA probe containing about 300 bp of the *sinI* promoter was mixed with a gradient of His_6_-ComK proteins in the mobility shift assay. ComK was found to shift the DNA fragment, indicating direct binding (upper panel, Fig. 6B). As a negative control, a similar size DNA probe containing a promoter of *ganS*, which is not known to be regulated by ComK (54, 55), was used in the same assay, and little DNA shift was observed (lower panel, Fig. 6B). Thus, the binding of ComK to the *sinI* promoter appeared to be specific.

To further test whether ComK recognizes the putative K-box in the *sinI* promoter, site-directed mutagenesis was performed on box 1 and box 3 of the putative K-box as indicated (Fig. 6C, nucleotide changes highlighted in red). Mutagenesis on box 2 and box 4 was avoided because of their overlap with the −35 and −10 promoter motifs (Fig. 6A). The reporter strains bearing P*_sinI_*-*lacZ* with site-directed mutations in the K-box were constructed, and the activities of those strains tested. The results showed that the point mutations in box 3 (mut2) and in both boxes 1 and 3 (mut1 + 2) had the greatest effect, resulting in increased *sinI* expression (Fig. 6D). Overall, the competence pathway negatively cross-regulates the biofilm pathway, likely through direct repression of the key biofilm regulatory gene *sinI* by the competence activator ComK.

### Biofilm matrix negatively impacts competence in *B. subtilis*.

**FIG 7:**
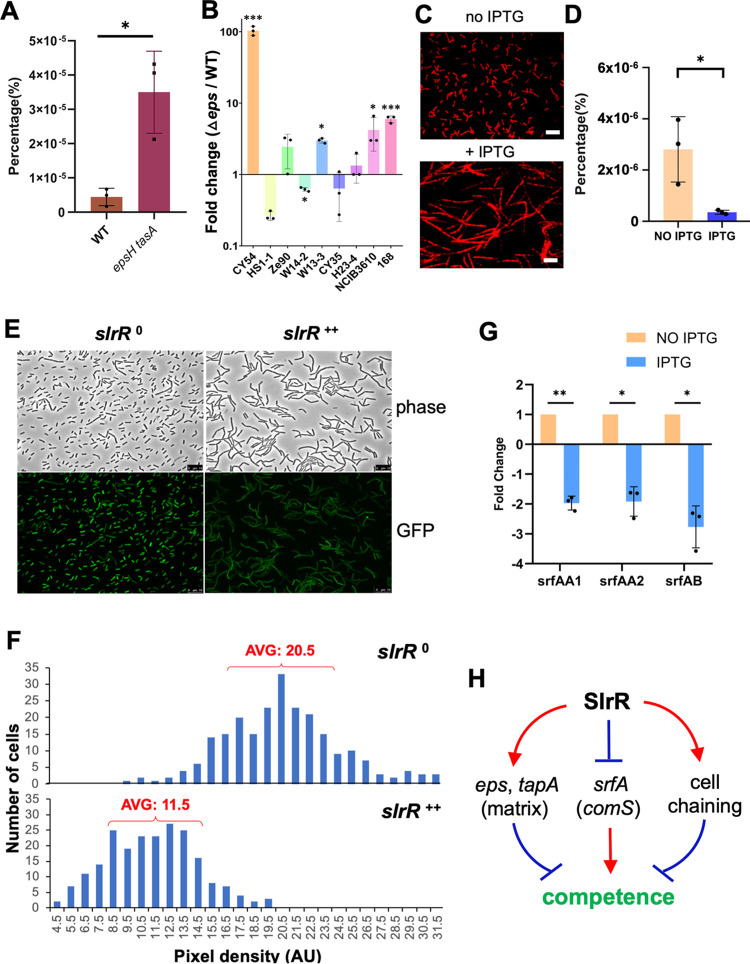
SlrR negatively regulates competence through three distinct mechanisms. (A) Comparison of the transformation efficiency of the wild type (strain 3610) and the Δ*epsH* Δ*tasA* double mutant (YC775). The results are presented as percentages of the number of transformants relative to the total number of cells. The experiment was repeated three times. Each dot indicates one biological replicate. Error bars indicate the standard deviation. *, *P* < 0.05. (B) Comparison of the transformation efficiency between the seven environmental strains of B. subtilis and their respective Δ*epsA-O* mutants (4). Strains 168 and 3610 and the Δ*epsA-O* mutants of strains 168 and 3610 were also included. The results are shown as the fold changes of CFU counts during transformation comparing the Δ*epsA-O* mutants and the respective wild-type strains. Each dot indicates one biological replicate. Error bars indicate the standard deviations. *, *P* < 0.05; ***, *P* < 0.0005. (C) Microscopic images of an *slrR*-inducible strain (YC672). Cells were grown in shaking LB medium without or with the addition of 100 μM IPTG to induce *slrR* expression and the cell chaining phenotype. Red indicates cell membrane staining by the membrane dye FM 4-64. Scale bars, 10 μm. (D) Comparison of the transformation efficiency of the *slrR*-inducible strain (YC672) in the absence or presence of 100 μM IPTG. The transformation efficiency is shown as the percentages of the numbers of transformants versus the total numbers of cells. The experiment was repeated three times. Each dot represents one biological replicate. Error bars indicate the standard deviations. *, *P* < 0.05. (E) Fluorescence microscopic analyses of the *slrR* overexpression strain harboring a fluorescent reporter of P*_srfAA_*-*gfp* (YC1270) in the absence or presence of IPTG to induce *slrR* expression. Cells were grown with shaking in MSgg to early log phase (OD_600_ = 0.3) and split into two fractions, one without IPTG (*slrR*^0^) and the other with 100 μM IPTG (*slrR*^++^) added to induce *slrR* expression for an hour before harvest and analysis of the cells. (F) Quantification of fluorescent pixel density of the cells in panel E by ImageJ (with the MicroJ plugin). More than 200 cells from each sample were randomly selected for analysis. The results are plotted indicating the difference in P*_srfAA_*-*gfp* activity without or with *slrR* overexpression. The numbers 20.5 (*slrR*^0^) and 11.5 (*slrR*^++^) indicate the average pixel densities (AU) of the top 50% of the cells in each population. (G) qPCR analyses were performed to test the negative regulation of SlrR on *srfAA-AD*. Total RNA was prepared from the *slrR*-inducible strain (YC672) grown with (*slrR*^++^) or without (*slrR*^0^) 100 μM IPTG. Three primer pairs, two for detection of *srfAA* and one for detection of *srfAB*, were applied. Each experiment was repeated three times. Each dot indicates one biological replicate. The error bars indicate the standard deviations. *, *P* < 0.05; **, *P* < 0.005. (H) Schematic drawing showing how the biofilm regulator SlrR negatively impacts competence through three distinct mechanisms. In Fig. 7, a *t* test was applied for statistical analysis.

We also predicted that the biofilm pathway negatively regulates competence development. A previous study showed that the extracellular matrix physically blocked cells from sensing the competence pheromones, which are essential for ComA-ComP-mediated activation of the *srfAA-AD* operon and *comS* (41, 49). Here, we further showed that deleting the biofilm matrix genes (Δ*epsH* Δ*tasA*) improved competence of B. subtilis 3610, with the transformation efficiency of the double mutant being ∼7-fold higher than that of the wild type (Fig. 7A). This phenomenon was not only seen in strain 3610 but also in some other environmental isolates of B. subtilis. When the *epsA-O* operon was deleted from these environmental isolates and the transformation efficiency of the mutants was compared to the respective wild-type strains, an increase in transformation efficiency from about 2- to 100-fold was seen in six of the nine strains (Fig. 7B; see also Fig. S3B). These results may suggest a general mechanism through which the presence of extracellular matrix can reduce the competence of B. subtilis cells.

### Extensive chaining in the biofilm negatively impacts competence.

Cells in the B. subtilis biofilm form long bundled chains, which are important for the organized three-dimensional structure of the biofilm (Fig. 4A) (19, 56). Interestingly, the DNA uptake machinery was shown to localize to the poles of the cells during competence development (57, 58). If true, one would predict that the nonmotile chained cells may encounter reduced efficiency at DNA uptake. To test the possible impact of chaining on competence, we first applied a Δ*sigD* mutant. SigD is a sigma factor responsible for the transcription of genes encoding multiple autolysins for cell separation but is not known to directly influence competence (59). The Δ*sigD* mutant formed extensive long chains (see Fig. S4A in the supplemental material) (59). When the transformation efficiency was compared between the wild type and the Δ*sigD* mutant, the mutant showed drastically reduced efficiency, even after the impact of cell chains on CFU counting was considered (see Materials and Methods). The diminished competence in the Δ*sigD* mutant was comparable to that of the Δ*comK* mutant (see Fig. S4B).

10.1128/mSystems.00539-20.4 FIG S4 Δ*sigD* cells form long chains and demonstrate very low transformation efficiency. (A) Microscopic images of the wild-type (strain 3610) cells and the Δ*sigD* mutant (RL4169), known to form long cell chains due to lack of SigD-controlled autolysin activities. Red indicates cell membrane staining by the membrane dye FM 4-64. Scale bar, 10 μm. The scale bar applies to both images here. (B) Comparison of transformation efficiency of the wild type (strain 3610) and the Δ*sigD* mutant (RL4169). The Δ*comK* mutant (YC100) is known to be deficient in transformation and was used as a negative control in the experiment. Assays were done in triplicates. Error bars represent the standard deviations. A *t* test was used to perform statistical analysis. **, *P* < 0.005. Download FIG S4, TIF file, 2.4 MB. Copyright © 2020 She et al. 2020 She et al. This content is distributed under the terms of the Creative Commons Attribution 4.0 International license.

In the wild-type biofilm, chaining is also controlled by the SinR/SlrR switch (26, 30, 32, 56, 59). SlrR^ON^ cells form long chains of cells bundled together by the extracellular matrix due to matrix genes being activated, while autolysin genes are simultaneously shut off (26). We decided to test whether increasing SlrR production by gene overexpression could have a similar negative impact on transformation efficiency. To accomplish this, a previously constructed Δ*slrR* mutant containing an IPTG-inducible copy of *slrR* was applied (56). The transformation efficiency of the engineered cells was compared between no addition and with the addition of IPTG. As shown in Fig. 7C and D, adding IPTG to the media significantly increased cell chaining, even under shaking conditions, while it substantially reduced the transformation efficiency compared to no addition of IPTG. Thus, our results suggest that extensive cell chaining during B. subtilis biofilm development likely plays a role in limiting the competence of B. subtilis cells.

### SlrR negatively regulates the *srfAA-AD* operon.

A previous study showed that Δ*sinR* diminished competence, but it was not clear how this occurred (60). Overproduction of the matrix by the Δ*sinR* cells may not have been the reason for the diminished competence since inhibition of competence by Δ*sinR* was reported in a laboratory strain that was unable to form robust biofilms. Here, we present evidence that SlrR, whose gene is repressed by SinR, negatively regulates *srfAA-AD* and thus competence (Fig. 1). We introduced a P*_srfAA_*-*gfp* fluorescent reporter into a *slrR* mutant bearing an IPTG-inducible copy of *slrR*. Upon the addition of IPTG, the activity of P*_srfAA_*-*gfp* noticeably decreased compared to no addition of IPTG (Fig. 7E). Quantification of the fluorescent pixel density in individual cells using ImageJ revealed that the average pixel density of cells overexpressing *slrR* was about half that of cells not overexpressing *slrR* (11.5 versus 20.5, Fig. 7F). The repression of *srfAA-AD* by SlrR was also confirmed by real-time quantitative PCR using three different probes for the operon (Fig. 7G). Thus, our results suggest that SlrR negatively regulates the *srfAA-AD* operon. In summary, we believe that SlrR, together with SinR, negatively regulates competence through three distinct mechanisms (Fig. 7H), by (i) promoting matrix production to block competence signaling, (ii) forming extensive cell chains to possibly block DNA uptake, and (iii) negatively regulating the *srfAA-AD* operon and *comS*.

## DISCUSSION

In this study, we found that during biofilm formation in the B. subtilis model strain 3610, a very low number of cells differentiate into competent cells. Similar observations were also made in several environmental strains of B. subtilis (Fig. 3). We presented evidence that competence is impacted by the ability of cells to form robust biofilms. The very low ratio of competent cells in the B. subtilis biofilm might be ecologically more relevant than what is often studied in the laboratory with optimized competence-promoting conditions. The biological implication of greatly reduced competence associated with biofilm formation by environmental B. subtilis strains is not very clear. One could argue that this is how natural competence in B. subtilis is expected to function to balance the ability to generate genetic variations and the potential risk of having too many individual cells in the population acquiring genetic variations.

Previous studies have demonstrated cell differentiation and coexistence of distinct cell types in the B. subtilis biofilm, and a few of these have characterized molecular mechanisms of how specific cell types become mutually exclusive or inclusive (2, 14, 26, 28, 29). In this study, we showed that competent cells and matrix producers are also mutually exclusive cell types during B. subtilis biofilm formation and provided mechanistic explanations of how they become mutually exclusive. Based on our evidence, we propose a working model of the cross-regulation between these two developmental pathways that contributes to their mutual exclusivity (Fig. 1). In one regulation, ComK in the K-state cells directly turns off the expression of the key biofilm gene *sinI*. Since the activity of *sinI* is indispensable for biofilm activation, repression of *sinI* by ComK allows K-state cells to shut down the biofilm pathway and eliminate matrix production and cell chaining, which we showed negatively influence transformation efficiency. In the other regulation, the biofilm regulator SlrR plays a central role in shutting down competence development. Not only may the matrix physically block competence quorum-sensing, but cell chaining and negative regulation of the *srfAA-AD* operon (and *comS*) by SlrR further contribute to the shutoff competence in SlrR^ON^ cells. Similar regulations may also be present in the B. subtilis environmental strains. However, since we primarily conducted our mechanistic studies in the model strain 3610, further studies will be needed to test those regulations directly in the environmental strains of B. subtilis. In addition, our data already indicate that the regulation in those environmental strains could differ from that in strain 3610 (e.g., Fig. 7B).

Previous studies have shown that competent cells are inclusive to those responding to the competence pheromones and activating the surfactin biosynthesis operon, which also transcribes a small gene, *comS*, essential to competence development (41, 49). According to a previous study, under biofilm conditions in strain 3610, the genes involved in initiating competence (*comQ-comX-comP*) are expressed in a majority of the cells, while the surfactin operon is expressed in only about 10% of the cells (defined as *srf*^ON^) (49). This difference in expression is thought to be contributed in part by the paracrine signaling mechanism in that, although the majority of cells produce the competence pheromone, only a handful respond to this quorum-sensing signal. Furthermore, another peptide pheromone, the competence and sporulation factor (CSF), is also involved by regulating the response regulator ComA through a feedback mechanism (61). It can be speculated that in the *srf*^ON^ cells, ComK is activated above a critical threshold in a further reduced ratio of the total cells because of the complex regulation of ComK and the *comK* gene. Those ComK^ON^ cells enter the so-called K-state and ultimately become competent for environmental DNA acquisition. It is a bit surprising that the ComK^ON^ cells (presumably cells expressing P*_comGA_*-*gfp*) comprised less than 0.1% of the total versus 10% of the cells being *srf*^ON^. This implies that, at best, only about 1 out of 100 cells enter the K-state, even after all the cells initiate the competence by inducing the *srfAA-AD* operon and *comS*. Again, it could be evolutionarily important to limit competence capacity when B. subtilis cells live in multicellular communities in the natural environment.

## MATERIALS AND METHODS

### Strains and media.

The strains used in this study are listed in Table 1. B. subtilis strains PY79, 168, NCIB3610, their derivatives, and environmental isolates of B. subtilis were cultured in lysogeny broth at 37°C. Pellicle biofilm formation in B. subtilis was induced using MSgg broth (50 mM potassium phosphate and 100 mM MOPS at pH 7.0 supplemented with 2 mM MgCl_2_, 700 μM CaCl_2_, 50 μM MnCl_2_, 50 μM FeCl_3_, 1 μM ZnCl_2_, 2 μM thiamine, 0.5% glycerol, and 0.5% glutamate) at 30°C. Colony biofilm formation of B. subtilis was induced using MSgg solidified with 1.5% (wt/vol) agar at 30°C. When growing the chromosomal *thrC* integration strains of B. subtilis, an additional 300 μg ml^−1^ threonine was added. Enzymes used in this study were purchased from New England Biolabs (Beverly, MA). Chemicals and reagents were purchased from Sigma or Fisher Scientific (Waltham, MA). Oligonucleotides were purchased from Eurofins Genomics (Pittsburgh, PA) and DNA sequencing was also performed at Eurofins Genomics. Antibiotics, if needed, were applied at the following concentrations: 5 μg ml^−1^ of tetracycline, 1 μg ml^−1^ of erythromycin, 100 μg ml^−1^ of spectinomycin, 10 μg ml^−1^ of kanamycin, and 5 μg ml^−1^ of chloramphenicol for transformation in B. subtilis and 100 μg ml^−1^ of ampicillin and 50 μg ml^−1^ of kanamycin for E. coli DH5α and BL21(DE3) strains.

**TABLE 1:** Strains and plasmids used in this study

Strain or plasmid	Genotype	Source or reference
Strains		
*B. subtilis*		
PY79	SPβ-cured laboratory strain of *B. subtilis* as a host for transformation	68
168	A laboratory strain of *B. subtilis* as a host for transformation	
NCIB3610	Undomesticated *B. subtilis* strain capable of biofilm formation	19
EH41	*sacA*::P*_tapA_*-*mKate2* in 3610; Kan^r^	This study
EH43	A dual fluorescent reporter strain of *sacA*::P*_tapA_-mKate2* and *amyE*::P*_comGA_-gfp* in 3610; Km^r^ Cm^r^	This study
EH44	*sacA*::P*_tapA_-mKate2*, *amyE*::P*_comGA_-gfp*, and *thrC*::*P_hpspank_-comK* in 3610; Km^r^ Cm^r^ Erm^r^	This study
RL4169	Δ*sigD*::*tet* in 3610; Tet^r^	59
YC100	Δ*comK*::*kan* in 3610; Km^r^	This study
YC108	*amyE*::*P_sinR_-lacZ* in 3610; Cm^r^	51
YC110	*amyE*::*P_sinI_*_(_*_WT_*_)_*-lacZ* in 3610; Cm^r^	51
YC130	*amyE*::*P_epsA_-lacZ* in 3610; Cm^r^	51
YC157	*amyE*::P*_hpspank_*-*comK* in 3610; Spec^r^	This study
YC159	*thrC*::*P_hpspank_-comK* and *amyE*::*P_sinI_-lacZ* in 3610; Erm^r^ Cm^r^	This study
YC160	*thrC*::*P_hpspank_-comK* and *amyE*::*P_epsA_-lacZ* in 3610; Erm^r^ Cm^r^	This study
YC177	*thrC*::*P_hpspank_-comK* and *amyE*::*P_sinR_-lacZ* in 3610; Erm^r^ Cm^r^	This study
YC672	Δ*slrR*::*tet* and *amyE*::*P_hpspank_-slrR*, in 3610; Tet^r^ Spec^r^	26
YC775	*ΔepsH*::*tet* and *ΔtasA*::*spec* in 3610; Tet^r^ Spec^r^	This study
YC1270	*amyE*::*P_hpspank_-slrR* and *lacA*::*P_srfAA_-gfp* in 3610; Spec^r^ Erm^r^	This study
QS34	*amyE*::P*_comGA_*-*gfp* in 3610, Cm^r^	This study
QS35	*thrC*::*P_spank_-comK* and *amyE*::*P_sinI_*_(_*_WT_*_)_*-lacZ* in 3610; MLS^r^ Cm^r^	This study
QS36	*thrC*::*P_spank_-comK* and *amyE*::*P_sinI_*_(_*_mut1_*_)_*-lacZ* in 3610; MLS^r^ Cm^r^	This study
QS37	*thrC*::*P_spank_-comK* and *amyE*::*P_sinI_*_(_*_mut2_*_)_*-lacZ* in 3610; MLS^r^ Cm^r^	This study
QS38	*thrC*::*P_spank_-comK* and *amyE*::*P_sinI_*_(_*_mut1+2_*_)_*-lacZ* in 3610; MLS^r^ Cm^r^	This study
QS42	*ΔdegQ*::*spec* in Ze90; Spec^r^	This study
QS43	*ΔdegQ*::*spec* in HS1-1; Spec^r^	This study
QS44	*ΔdegQ*::*spec* in CY35; Spec^r^	This study
QS45	*ΔdegQ*::*spec* in CY54; Spec^r^	This study
QS46	*ΔdegQ*::*spec* in W13-3; Spec^r^	This study
QS47	*ΔdegQ*::*spec* in W14-2; Spec^r^	This study
QS48	*ΔdegQ*::*spec* in H23-4; Spec^r^	This study
CY35	An environmental isolate of *B. subtilis*	4
CY54	An environmental isolate of *B. subtilis*	4
H23-4	An environmental isolate of *B. subtilis*	4
HS1-1	An environmental isolate of *B. subtilis*	4
W13-3	An environmental isolate of *B. subtilis*	4
W14-2	An environmental isolate of *B. subtilis*	4
Ze90	An environmental isolate of *B. subtilis*	4
CY94	*ΔdegU* in 3610; Tet^r^	This study
CY96	*ΔdegS* in 3610; Tet^r^	This study
CY601	*ΔdegQ*::*spec* in 3610; Spec^r^	This study
CY602	*degQ* overexpression in 3610; Spec^r^	This study
*E. coli*		
DH5α	An *E. coli* host for molecular cloning	Invitrogen
QS16	*E*. *coli* BL21(DE3) with plasmid pQS06	This study
		
Plasmids		
pYC119	*amyE*::P*_hpspank_*-*comK* in pDR111; Amp^r^ Spec^r^	This study
pYC121	*amyE*::*gfp* (promoterless) in pDG1662; Amp^r^ Sm^r^	51
pYC128	*thrC*::P*_hpspank_*-*comK* in pDG1664; Amp^r^ Erm^r^	This study
pYC166	*amyE*::P*_sinI_*-*lacZ* in pDG268; Amp^r^ Cm^r^	33
pQS06	pET28a(P_T7_-*his6-comK*) plasmid; Kan^r^	This study

### Strain construction and DNA manipulation.

General methods for molecular cloning followed the published protocols (62). Restriction enzymes (New England Biolabs) were used according to the manufacturer’s instructions. Transformation of plasmid DNA into B. subtilis strains was performed as described previously (63). SPP1 phage-mediated general transduction was also used to transfer antibiotic-marked DNA fragments among different strains (64). Plasmids used in this study are listed in Table 1, and oligonucleotides are listed in Table 2.

**TABLE 2:** Oligonucleotides used in this study

Primer	Sequence (5′–3′)^*a*^
comK-F1	gact aagctt aaggatggaggccataatatg
comK-R1	gact gctagc ctaataccgttccccgagctc
PcomK-F1	gtc catatg agtcagaaaacagacgca
PcomK-R1	gtc aagctt ctaataccgttccccgag
PsinI_Mut1_-F	acttttttaccattcgaca ** gtg ** ttctcgttttttttgagaa
PsinI_Mut1_-R	ttctcaaaaaaaacgagaa ** cac ** tgtcgaatggtaaaaaagt
PsinI_Mut2_-F	tcgttttttttgag ** tcg ** atacgattataataa
PsinI_Mut2_-R	ttattataatcgtat ** cga ** ctcaaaaaaaacga
PsinI-F	agaaaaacaggcgctgaaaa
PsinI-R	cagtccggccatgacttatt
PsinI-F4	gtca gaattc tttcactgacgtctca
PsinI-R4	gtca aagctt ctcctcctaaaatactt
PganS-F	gtac gaattc cggacccgattgcagtgggctg
PganS-R	gtac ggatcc ttcggtaggaatgaaagcgct
PcomGA-F1	gtac gaattc tcttgaaaatgaccaaatgaccgg
PcomGA-R1	sgtac aagctt caacgcatattgtagaaaaagaagaaaagg
PdegQ-F	tcggtagaacgaaaaaaaagacttg
PdegQ-R	aaacgctctttcgcatagaaagata
srfAA1F	ctttttaccctttaacggatgcaca
srfAA1R	gttttcatctagccgcaaccgaagg
srfAA2F	tttacgcaaatgttcatcacgtgat
srfAA2R	ttcttttgtctctgagccgctggct
srfAB1F	cacaattagagcttgggattcacggc
SrfAB1R	ctgatgcacaaataccgtacggaga

a The underlined letters indicate restriction enzyme recognition sequences. The boldfacing in the sequences indicates nucleotide changes for site-directed mutagenesis.

To generate the competence gene reporter strains (P*_comGA_*-*gfp*), the promoter of *comGA* was amplified via PCR using strain 3610 genomic DNA as the template and primers PcomGA-F1 and PcomGA-R1. The PCR product was cloned into the EcoRI and HindIII sites of pYC121 aiming for the integration into the *amyE* locus of strain 3610 and other environmental isolates of B. subtilis. The recombinant plasmid was transformed into DH5α for amplification. The recombinant plasmid extracted from transformed DH5α was subsequently transformed into PY79 and then to strain 3610.

To generate *comK* insertional deletion mutation in strain 3610 (YC100), the lysate containing Δ*comK*::*kan* was made from RL2262 (a gift from Rich Losick, Harvard University) and introduced into strain 3610 by transduction. To create an IPTG-inducible copy of *comK* for integration at the *amyE* locus, the *comK* coding sequence was amplified by PCR using the primers comK-F1 (HindIII) and comK-R1 (NheI). The PCR product was digested and cloned into the HindIII and NheI sites of pDR111 (65) to make an IPTG-inducible P*_spank_*-*comK* fusion, generating the recombinant plasmid pYC119. The pYC119 plasmid was then used for integration of P*_spank_*-*comK* into the *amyE* locus of strain 3610. To do so, the plasmid was first introduced into PY79 by transformation and then into 3610 by SPP1 phage mediated transduction. To create a second version of an IPTG-inducible *comK* for integration at the *thrC* locus of strain 3610, a DNA fragment containing the P*_spank_* promoter was cut from the above pYC119 with EcoRI and HindIII double digestion, and a second DNA fragment containing the *comK* coding sequence and the *lacI* gene was cut separately from pYC119 by HindIII and BamHI double digestion. These two DNA fragments were cloned into the EcoRI and BamHI sites of pDG1664 by three-way ligation to generate an IPTG-inducible P*_spank_*-*comK* in the *thrC* integration plasmid, resulting in pYC128. The pYC128 plasmid was introduced into PY79 by transformation. To generate three reporter strains of YC159 (P*_sinI_*-*lacZ*), YC160 (P*_epsA_*-*lacZ*), and YC177 (P*_sinR_*-*lacZ*), each with an inducible copy of *comK* at the *thrC* locus, lysate containing *thrC*::P*_spank_*-*comK*::*mls* was prepared from the recombinant pY79 strain described above and introduced into YC108 (P*_sinR_*-*lacZ*), YC110 (P*_sinI_*-*lacZ*), and YC130 (P*_epsA_*-*lacZ*), respectively, by SPP1 phage-mediated transduction.

To generate the recombinant plasmid pQS06 for His_6_-ComK overexpression and purification, the *comK* coding gene was amplified by PCR using 3610 genomic DNA as the template and primers PcomK-F1 and PcomK-R1. The PCR product was cloned into the pET28a vector between the restriction sites NdeI and HindIII to create the PT7-his6-*comK* fusion. The recombinant plasmid pQS06 was prepared from E. coli DH5α and then introduced into E. coli BL21(DE3) by chemical transformation. The resulting E. coli strain QS16 was used for His6-ComK overexpression and purification. To create strain YC1270, lysate containing *amyE*::P*_hyperspank_*-*slrR* was prepared from YC672 and introduced into DL744, which bears the reporter *lacA*::P*_srfAA_*-*gfp*::*mls*, by transduction (49, 56). To create the transcription reporter fusion of P*_comGA_*-*gfp*, the promoter sequence of the *comGA* gene was amplified by PCR using strain 3610 genomic DNA as the template and primers PcomGA-F1 and PcomGA-R1. The PCR product was cloned into the pYC121 plasmid between the restriction sites EcoRI and HindIII to create the P*_comGA_*-*gfp* fusion. The recombinant plasmid was transformed into DH5α for amplification. The recombinant plasmid extracted from transformed DH5α was subsequently transformed into PY79 and then to strain 3610.

### Colony and pellicle biofilm development.

For colony biofilm formation, cells were grown to exponential phase in Luria-Bertani (LB) broth and 2 μl of the culture was spotted onto MSgg media solidified with 1.5% (wt/vol) agar. The plates were incubated at 30°C for 3 days. For pellicle biofilm formation, cells were grown to exponential phase in LB broth, and 3 μl of the culture was inoculated into 3 ml of MSgg liquid media in a 6-well or 12-well microtiter plate (VWR). The plates were incubated at 30°C for 2 to 3 days. Images of colony and pellicle biofilms were taken using a Nikon Coolpix camera or a Leica MSV269 dissecting scope.

### Site-directed mutagenesis.

Site-directed mutagenesis of the *sinI* regulatory sequence was performed by using six different primers to change the nucleotides in the putative ComK binding boxes in the *sinI* promoter region. In the P*_sinI_*^Mut1^ construction (box 1), PsinI-F4 and PsinI^Mut1^-R were used to amplify the fragment 1. PsinI-R4 and PsinI^Mut1^-F were used for the amplification of fragment 2. The fragments 1 and 2 were subsequently used in the second round of overlapping PCR to generate the full-length DNA fragment containing the *sinI* promoter with designated point mutations. Overlapping PCR product was purified using PCR purification kit (Qiagen) and subsequently digested using EcoRI and HindIII. The plasmid pDG268 was digested simultaneously using EcoRI and HindIII. Both digestion products were gel-purified, ligated using T4 ligase, and transformed E. coli DH5α. The recombinant plasmid was purified from E. coli DH5α and transformed into PY79. The *amyE* homologous region containing mutated P*_sinI_*^Mut1^ and the chloramphenicol resistance marker was integrated onto PY79 chromosome via double-crossover homologous recombination. The genomic DNA of the resulting transformant was prepared and subsequently transformed into strain 3610. site-directed mutagenesis on P*_sinI_*^Mut2^ (box3) was performed similarly, except that the primers PsinIMut2-F, PsinIMut2-R, PsinI-F4, and PsinI-R4 were used. Construction of P*_sinI_*^Mut1+2^ was performed by using the recombinant plasmid containing P*_sinI_*^Mut1^ as the template during the first round of PCR amplification and the primers PsinIMut2-F, PsinIMut2-R, PsinI-F4, and PsinI-R4. Designated point mutations in the *sinI* promoter on the recombinant plasmids were verified by DNA sequencing before being introduced into B. subtilis.

### Assays of transformation efficiency.

Assays on transformation efficiency were performed by introducing B. subtilis genomic DNAs containing specific antibiotic resistance genes as a selection marker into indicated strains. Specifically, the three plasmids pDG1662 (*amyE*::Chl^r^), pDG1663 (*thrC*::MLS^r^), and pDG1730 (*amyE*::Spec^r^) containing different antibiotic markers flanked by the either B. subtilis *amyE* or *thrC* sequences (26), were introduced into strain 3610 first for double-crossover recombination on the chromosome. The genomic DNA bearing either *amyE*::Chl^r^, or *amyE*::Chl^r^, or *thrC*::MLS^r^ was prepared from the strains described above. The concentration of the prepared genomic DNAs was determined using NanoDrop (Thermo Fisher). For each transformation event, a fresh single colony of the strain was picked and grown in LB broth to log phase. The log phase culture was then 1:100 subcultured into 2 ml of competence medium (MC) supplemented with 3 mM MgSO_4_. Cells were grown at 37°C with shaking until early stationary phase (optical density at 600 nm [OD_600_] = 1.5); 10 μg of the genomic DNA was then mixed with 500 μl of competent cells, and the cells were incubated for another hour before harvest. Samples were plated on the LB plates with the addition of either 100 μg/ml of spectinomycin (for Spec^r^ selection) or 5 μg/ml of chloramphenicol (for Chl^r^ selection) or 25 μg/ml of lincomycin plus 1 μg/ml of erythromycin (for MLS^r^ selection). The next day, the CFU on the transformation plates were counted. The total number of cells was calculated by measuring the OD_600_ of the culture prior to plating and assuming 3 × 10^8^ cells for an OD_600_ of 1.0 of the culture (which was experimentally determined for strain 3610 [data not shown]) across all B. subtilis cultures used in the transformation assays unless for the strains involving extensive cell chains (see below). Each assay was performed at least three times.

For transformation of the *slrR*-inducible strain (YC672), cells were grown in LB broth to log phase, diluted 1:100 into MSgg broth, and grown to mid-log phase (OD_600_ = 0.5) again. Cells were then split into two fractions; one added with 100 μM IPTG to induce *slrR* overexpression, and for the other there was no addition of IPTG. Both cultures continued to grow for another hour for *slrR* induction. Then, 10 μg of genomic DNA was added to each culture of 500 μl, followed by one more hour growth at 37°C with shaking. Before harvesting, the cultures were mildly sonicated (scale 1.5 output, 50% interval, three to five pulses on ice) using a sonicator (Scienz). After sonication, the cells were analyzed under light microbiology to verify the disruption of chaining. The cells were then plated on LB plates supplemented with appropriate antibiotics. The next day, the numbers of transformants on the plates were calculated. To count the total number of cells, cultures were serially diluted and plated on regular LB plates. The CFU were counted next day. All assays were performed at least three times with biological replicates. The transformation experiment using the *sigD* mutant followed a similar protocol to eliminate the impact of chaining.

### Expression and purification of recombinant ComK proteins.

BL21(DE3) cells harboring the recombinant plasmid (PT7-his6-*comK*) were grown in LB broth supplemented with 50 μg/ml kanamycin at 37°C overnight with shaking. The overnight culture was aliquoted at 1:500 to 300 ml of LB media supplemented with 50 μg/ml kanamycin with shaking at 30°C. Then, 1 mM IPTG was added when the OD_600_ of the culture reached 0.5. IPTG induction continued for 2 h before the culture was harvested. The culture was harvested and centrifuged at 4,500 rpm at 4°C for 30 min. The cell pellet was resuspended and washed twice using cold phosphate buffer solution. The supernatant was discarded, and the cell pellets were again resuspended using 10 ml of lysis buffer (20 mM Tris-HCl, 300 mM NaCl, 1 mM phenylmethylsulfonyl fluoride [pH 8.5]). The cell resuspension was lysed using sonication on ice. The total cell lysate was centrifuged at 5,000 rpm at 4°C for 30 min. The cleared lysate containing soluble His6-ComK was transferred into a new precooled tube. The cell lysate was mixed with 1 ml of Ni-NTA agarose beads (Qiagen), and the mixture was rotated at 4°C for 2 h. The mixture of lysate and beads was transferred into the column and washed five times with wash buffer (20 mM Tris-HCl, 300 mM NaCl, 25 mM imidazole [pH 8.5]). Next, 2 ml of wash buffer was applied for each wash. The flowthrough was also collected in five separate tubes. The column was eluted five times using elution buffer (20 mM Tris-HCl, 300 mM NaCl, 250 mM imidazole [pH 8.5]). Then, 500 μl of elution buffer was applied to the column each time, and the elute was collected in the tubes separately. Next, 12% SDS-PAGE was applied to size fractionate the proteins and verify the purity and abundance of the recombinant His6-ComK proteins. The purified protein fractions were pooled and dialyzed in a dialysis buffer (20 mM sodium phosphate, 300 mM NaCl, 0.3 mM dithiothreitol, 10% glycerol [pH 7.4]) overnight. The final concentration of the protein was determined by using Bradford protein assays. The proteins were stored at –80°C.

### Electrophoretic mobility shift assays.

An ∼300-bp DNA fragment containing the *sinI* promoter (P*_sinI_*) was used as the DNA probe for the binding of recombinant ComK proteins in an EMSA, and a similar-size DNA fragment containing the *ganS* promoter (P*_ganS_*) was used as a negative-control DNA probe. The fluorescent DNA probes were generated by PCR using strain 3610 genomic DNA as the template and with the forward primers P*_sinI_*-F and P*_ganS_*-F covalently linked to 5′Cy3 fluorescent dye and the regular reverse primers P*_sinI_*-R and P*_ganS_*-R. The PCR product was gel purified and eluted in ddH_2_O, and the quality was measured by using a NanoDrop apparatus (Fisher Thermo Scientific). A gradient of protein concentrations was applied in the reaction mixtures. A decreasing gradient of 150, 60, 15, and 7.5 nM recombinant His_6_-ComK was applied in each binding mixture. Then, 200 pmol of fluorescent labeled DNA probe was applied in each lane. The protein-DNA binding reaction mixture was incubated in a 20-μl reaction volume containing 10 mM Tris-HCl, 10 mM HEPES, 50 mM KCl, 1 mM EDTA, 10 μg/ml bovine serum albumin, and 4% sucrose. To reduce nonspecific binding, 500 ng of random DNA [poly(dI-dC)] was added to each binding reaction. The reaction mixture was incubated on ice for 30 min. The gel was run in 0.5× Tris-borate-EDTA buffer at 65 V for 3.5 h at 4°C. The resulting gel was imaged using ChemiDoc MP (Bio-Rad).

### Assays of β-galactosidase activity.

Cells were cultured in MSgg medium at 30°C with shaking. When indicated, IPTG was added to the media at the beginning at a final concentration of 10 μM. Next, 1 ml of culture was collected at each indicated time point, and the cells were centrifuged down at 5,000 rpm for 10 min. Cell pellets were suspended in 1 ml of Z buffer (40 mM NaH_2_PO_4_, 60 mM Na_2_HPO_4_, 1 mM MgSO_4_, 10 mM KCl, and 38 mM β-mercaptoethanol) supplemented with 200 μg ml^−1^ lysozyme. Resuspensions were incubated at 37°C for 15 min. Reactions were started by adding 200 μl of 4 mg ml^−1^ ONPG (2-nitrophenyl-β-d-galactopyranoside) and stopped by adding 500 μl of 1 M Na_2_CO_3_. The samples were then briefly centrifuged down at 5,000 rpm for 1 min. The soluble fractions were transferred to cuvettes (VWR), and the absorbance of the samples at 420 nm was recorded using a Bio-Rad spectrophotometer. The β-galactosidase specific activity was calculated according to the following equation: (*A*_420_/time × OD_600_) × dilution factor × 1,000. Assays were conducted in triplicate.

### Cell membrane staining.

For cell membrane staining, cells were grown to log phase and harvested. Cell pellets were washed with phosphate-buffered saline (PBS) twice, resuspended in 100 μl of PBS, and mixed with 1 μl of FM 4-64 dye (Life Technologies) for 5 min on ice with gentle tapping of the tube. Then, 2 μl of the resuspension was placed on a 1% (wt/vol) agarose pad and covered with a coverslip. To observe the FM 4-64 fluorescence dye, the excitation wavelength was set at 540 to 580 nm and the emission wavelength at 610 to 680 nm. Cells from three independent biological replicates were imaged using a Leica DFC3000 G camera on a Leica AF6000 microscope.

### Real-time quantitative PCR.

Cells were collected after the overexpression of SlrR in an experimental group. Total RNAs were extracted by using TRIzol (Invitrogen) according to the manufacturer’s protocol. Isolated RNAs were reverse transcribed into single-stranded complementary DNA (cDNA) using a high-capacity cDNA reverse transcription kit (Applied Biosystems). Real-time quantitative PCR (RT-qPCR) was performed by using Fast SYBR Green Master Mix (Applied Biosystems) with a Step-One Plus real-time PCR system (Applied Biosystems). The 16S rRNA gene was used as an internal reference. The relative expression of specific genes was calculated by using the 2^−ΔΔ^*^CT^* method. A *t* test was performed to determine statistical significance.

### Cell fluorescence imaging and pixel quantification.

To image the environmental strains bearing the P*_comGA_-gfp* fluorescent reporter, the reporter strains were grown in LB broth to log phase. Each log-phase culture was then 1:100 subcultured into 2 ml of competence medium (MC) supplemented with 3 mM MgSO_4_. The cells were grown at 37°C with shaking until reaching early stationary phase (OD_600_ = 1.5). They were then spun down, washed with PBS once, and resuspended in 100 μl of PBS. Next, 2 μl of the resuspension was placed on a 1% (wt/vol) agarose pad, covered with a cover slip, and observed using fluorescence microscopy. Imaging of different samples was conducted using the same exposure settings.

To quantify the ratio of P*_comGA_-gfp*-expressing cells relative to the total number of cells, the fluorescence of single cells was quantified in three different images comprising of a total of 600 to 800 cells per sample using the MicrobeJ plugin for ImageJ (66, 67). These three images were randomly selected from more than a half-dozen separate images obtained in two experimental repeats. Using the “analyze particles” command, a size cutoff of 100 square pixels was set to exclude the noise from the viable cells. Using the “threshold” command, the threshold number was adjusted to highlight and select the pixel area of interest, which indicates the viable cells, for the analysis. A threshold above three times of the average background pixel density was used to define P*_comGA_-gfp*-expressing cells. The total numbers of cells were counted in phase images, while the fluorescent cells were counted with the corresponding fluorescent channel images and verified in combination with manual examination.

To image the *slrR*-inducible strain (YC1270), the cells were grown in LB broth to log phase. The cells were then diluted 1:100 to MSgg broth and grown to mid-log phase (OD_600_ = 0.5). The cells were then split into two fractions, one with 100 μM IPTG added and the other with no IPTG added. Both fractions were continued to grow for another hour. The cells were then spun down, washed with PBS once, and resuspended in 100 μl of PBS. Next, 2 μl of the resuspension was placed on a 1% (wt/vol) agarose pad, covered with a coverslip, and observed using fluorescence microscopy. The nonspecific background fluorescence was determined by quantifying wild-type cells bearing no fluorescent reporter. Imaging of different samples was conducted at the same exposure settings. The pixel density of single-cell fluorescence was quantified on >200 cells per sample using the MicrobeJ plugin for ImageJ.

### Flow cytometry.

Flow cytometry was carried out using a BD FACSAria II with a 70-μm nozzle. Briefly, biofilms were grown for 48 h in defined monosodium glutamate-glycerol (MSgg) biofilm-promoting media. After 48 h of growth, the cells were harvested from pellicle biofilms, the cell chains were disrupted by mild sonication, and 5-μl portions of resuspended cells were diluted in 1 ml of PBS through a 35-μm filter (Corning Falcon tube; Thermo Fisher). FACSDiva software was used to collect 100,000 events for each sample. The data were analyzed in FlowJo software. Gates were drawn, based on the size, to exclude the bulky events, which were considered as clumps or cell chains. This size bias was confirmed by plotting these events on the FSC/SSC axis. The rest of the gated cells were considered to be single cells and displayed in GFP-A/mKate-A axis. Four strains—strain 3610 as a gating control for fluorescent signals, two single reporter strains, (P*_comGA_*-*gfp* and P*_tapA_*-*mkate2*), and the dual reporter strain (P*_comGA_*-*gfp*/P*_tapA_*-*mkate2*)—were applied in the analyses by flow cytometry. Assays were performed in three biological replicates.
