# Supplementary figures and images for: Negative Interplay between Biofilm Formation and Competence in the Environmental Strains of Bacillus subtilis
Source: mSystems. 2020 Sep 1;5(5):e00539-20. doi: 10.1128/mSystems.00539-20 (PMC7470987; doi:10.1128/mSystems.00539-20)

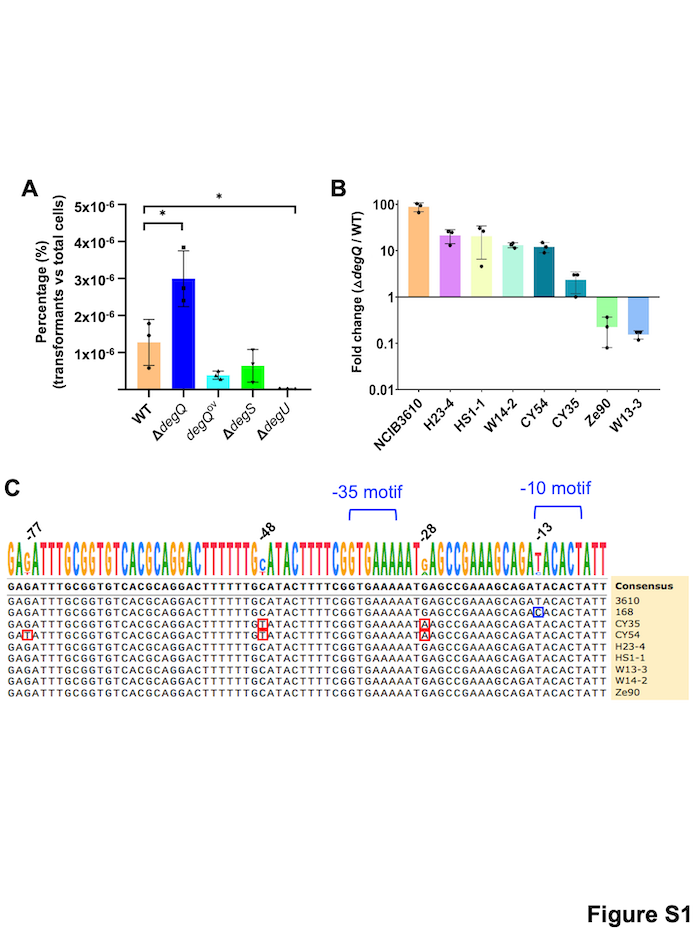

Supplement: FIG S1 [file mSystems.00539-20-sf001.tif]

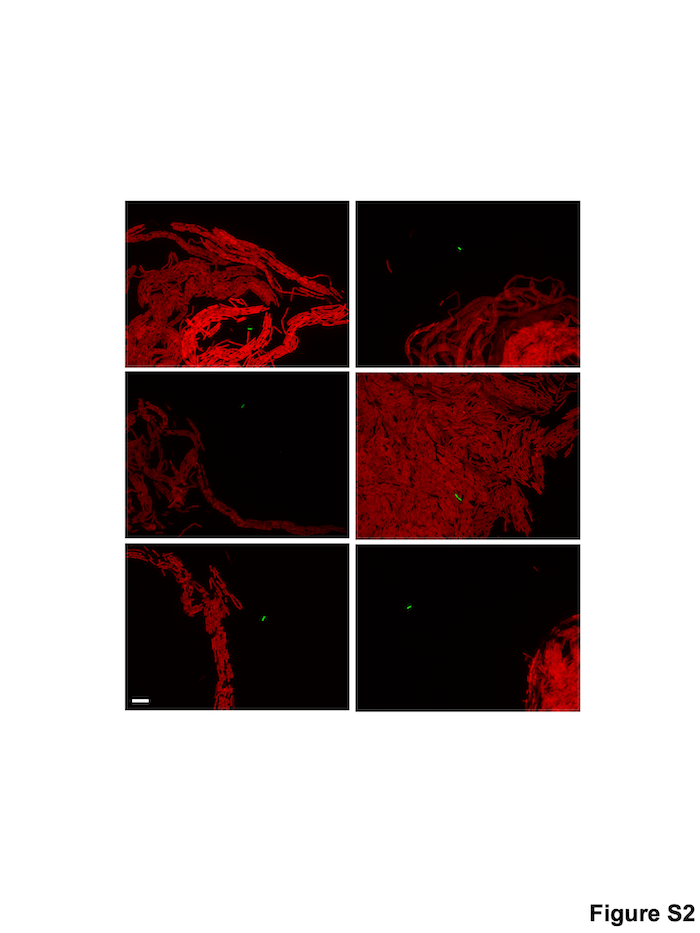

Supplement: FIG S2 [file mSystems.00539-20-sf002.tif]

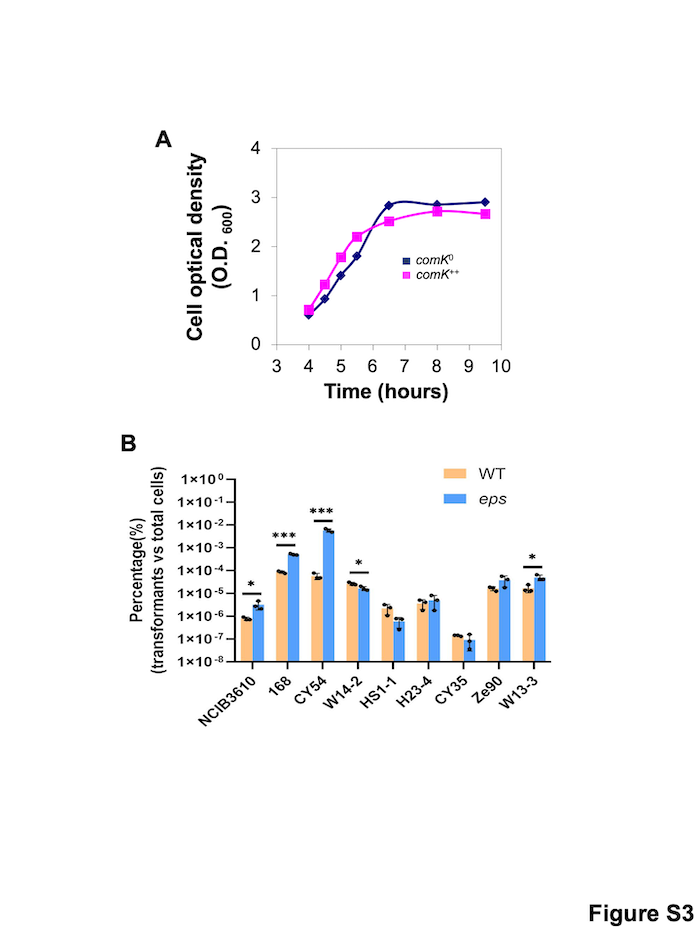

Supplement: FIG S3 [file mSystems.00539-20-sf003.tif]

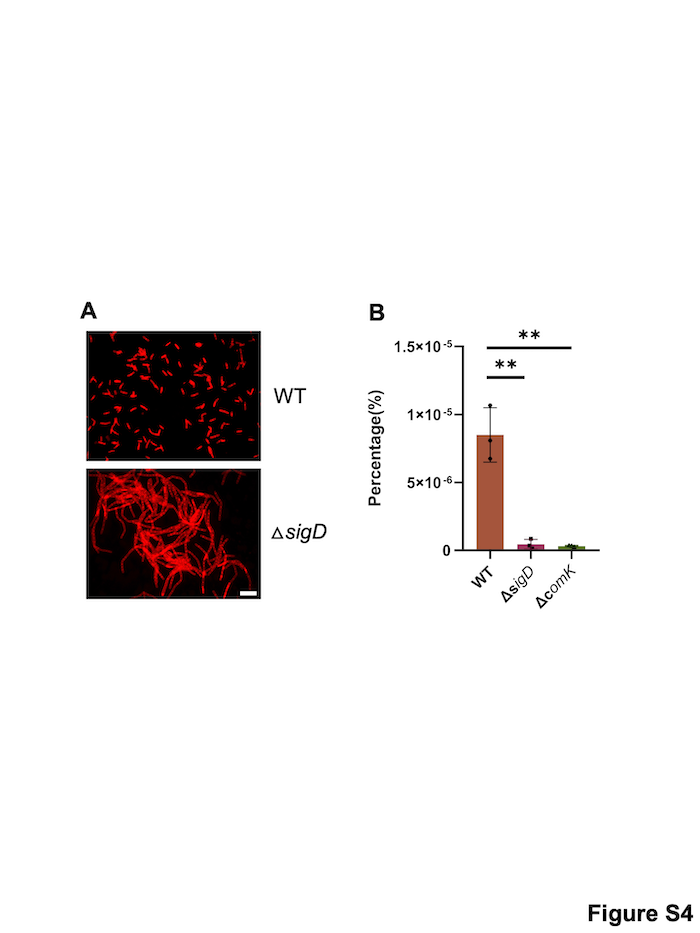

Supplement: FIG S4 [file mSystems.00539-20-sf004.tif]
